# The Accumulation and Transformation of Heavy Metals in Sediments of Liujiang River Basin in Southern China and Their Threatening on Water Security

**DOI:** 10.3390/ijerph19031619

**Published:** 2022-01-31

**Authors:** Xiongyi Miao, Mian Song, Gaohai Xu, Yupei Hao, Hucai Zhang

**Affiliations:** 1 Key Laboratory of Karst Dynamics, MNR&GZAR, Institute of Krast Geology, Chinese Academy of Geological Sciences, Guilin 541004, China; miaoxy88@126.com; 2 Department of Health Management, Guiyang Healthcare Vocational University, Guiyang 550001, China; 3 Henan Xinweijie Technology Co., Ltd., Luoyang 471000, China; 4 Institute for Ecological Research and Pollution Control of Plateau Lakes, School of Ecology and Environmental Science, Yunnan University, Kunming 650500, China; 5 Center for Hydrogeology and Environmental Geology, CGS, Baoding 071051, China; songmian88@126.com; 6 Nanjiang Hydrogeological & Engineering Geology Brigade, Chongqing Bureau of Geology and Minerals Exploration, Chongqing 401121, China; ccqxgh@163.com

**Keywords:** Liujiang River, heavy metals, speciation, risk assessment

## Abstract

Heavy metal (HM) pollution in sediments is tightly related to the security of water quality in rivers, but the accumulation and conversion of HMs are poorly researched, so that a field study was conducted as an example in the Liujiang River Basin. Seven HMs were analyzed to determine between the overlying water and sediments. Moreover, the regulation of HMs speciation and environmental factors in their accumulation and conversion were identified. The obtained results suggested the HM concentrations in water are far below the primary standard of water quality, but in sediments, the contents of Cd and Zn are significantly higher than their corresponding baseline of soil. Only Cd and Pb are dominantly in non-residual form (carbonate-bound fraction and reducible fraction, respectively). The non-significant correlations suggested pH and Eh may be hard to influence HMs in water, while the significant correlations highlighted the regulations of Eh, organic matter and mean grain size on the accumulation of metals in sediments. The opposite correlations between EC, TDS, pH and Cd confirmed the emission of acid wastewater contributed to the accumulation of Cd in sediment. The conversion of metals between water and sediments were found to be significant only in specific forms of Cd, As, Cu, Zn and Pb, suggesting the conversion of HMs in sediments should be largely regulated by their specific forms. The very high risk disclosed by the higher values of $E_{r}^{i}$ and RI are only found upstream, while the higher risk of Cd should be treated as a critical environmental threat.

## 1. Introduction

Rivers are vital channels of water resource regulation and storage; however, with the development of industry and agriculture, they have gradually become an important channel for sewage discharging. Based on the previous investigations, the contamination of HMs with various degrees could be commonly found in rivers all over the world, indicating a severe situation of HMs pollution in rivers [1,2,3,4,5]. Therefore, the strict control of sewage discharging has been proposed to protect the ecological environment of rivers. The improvement of water quality in rivers actually confirmed the positive influences of wastewater regulation on degrading the environmental threat of HMs, but the HM pollution among aquatic biotas in rivers still suggested that the threat of HMs in rivers cannot be eliminated by only relying on the regulation of wastewater discharge [6,7,8,9]. In fact, a previous study indicated that the bio-enrichment of HMs among aquatic biotas is less susceptible to the HMs in surface water, despite the dissolved HMs having higher bioavailability, on the contrary, the sediments with massive exogenous HM aggregation can play a critical role in HM bioaccumulation among aquatic biotas indeed [10,11,12]. As is known to all, the threat of HMs mainly results from HM bioaccumulation among biotas, which would damage the health of biotas and humans. Therefore, it is of great value to deal with HMs in sediments, instead of only regulating wastewaters discharging, for degrading the threat of HM pollution in rivers.

HMs exist in sediments in a variety of chemical forms, they can be combined with carbonate, Fe/Mn oxy-hydroxide, sulfide, organic matter and other active substances [13,14,15]. To simplify the existence forms of HMs, researchers proposed multiple analysis methods [16,17,18,19]. The most widely used method was proposed by Tessier, which divided HMs into exchangeable, carbonate-bound, reducible, oxidizable and residual fractions [17]. In the view that metals in residual phase are considered to be protogenetic in original minerals, which is relatively inert and difficult to exert ecological effects, exogenous metals could be only reserved as a weakly bonded form in these non-residual forms, the forms of which were also considered to be the critical supporter on the conversion of HMs [20,21,22,23]. As is known to all, the composition of metals speciation in sediments is not invariant; the composition of metals speciation should be varied with the accumulation of metals inevitably. However, the interactions between metal accumulation and metal speciation were rarely detailed in the previous study, which did not determine the dominant forms of metals that back up metal accumulation in sediments. Given the accumulation of metals is susceptible to environmental changing, despite Eh, pH, temperature, salinity, granularity, photosynthesis, the strength of metallic ion and organic matter, etc., can all manipulate the transformation of HMs in the sediments [24,25,26,27,28,29], the dominant environmental factors that can regulate the accumulation and conversion of HMs still need to be ascertained, which is of great value to the treatment of metal contamination in rivers.

The Liujiang River Basin is located in the southwest of China, governed by Liuzhou City, which is geographically the second largest city and the largest industrial city in Guangxi Province with the integrated industrial system. The extensive industrial operations have made Liuzhou a key city with huge discharge of effluent. The annual emission of wastewater is higher than 350 million tons, more than 80% of which comes from industrial operations related to metal smelting, chemicals, foods and paper industries [6]. The wastewater containing metals in high content was largely discharged into the waterways of Liuzhou City and it eventually converges into the Liujiang River and its tributary the Luoqingjiang River, which are the most important surface runoffs of Liujiang River Basin. However, with the shield of the carbonate ions in karst river [30], exogenous metals did not appear in water with large amounts, which confirmed by the significantly low content of metals within threshold in water [31]. On the contrary, the high ratio of metals in non-residual form reported formerly, suggested exogenous metals could largely hidden in sediments [32]. Despite the composition of metals speciations in sediments were investigated formerly in Liujiang River, the interactions were only referred to be between metals bioavailability and their chemical forms [30,32]. Their interactions on metals accumulation and conversion have not been identified. With the high ratio of metals in non-residual forms, the accumulation of metals may be more likely to be impaired with environmental fluctuations, then aggravate the conversion of metals between water and sediments, which, however, undoubtedly should be treated as a great threat to the security of water quality in Liujiang River. The security of water quality in Liujiang River completely depends on the interactions between environmental fluctuations and metals speciation, and it is essential to identify the interactions between environmental fluctuations and metals speciation, which should be not only help to detail the accumulation and conversion of metals in the Liujiang River, but also improve the management of metal contamination in the Liujiang River. The focus of our study is: (1) To determine the variation of seven HMs (i.e., Zn, Cd, As, Pb, Cr, Cu, and Hg) between the overlying water and sediment in Liujiang River Basin; (2) to detail the roles of HM speciation and physicochemical property of water and sediments on their accumulation and conversion of HMs; and (3) to assess the potential ecological risks of seven HMs in surface sediments.

## 2. Materials and Methods

### 2.1. The Description of Study Area and Field Sampling

The Liujiang River Basin is located in Guangxi Province, China with an area of 58,398 ${\mathrm{km}}^{2}$. The Liujiang River and Luoqingjiang River are the most important surface runoffs in this watershed. Liujiang River is the largest river in Liuzhou City and the second largest tributary of the Xijiang River in the Pearl River Basin with a total length of 272 km, which stems from the confluence of Longjiang and Duliujiang River in Fengshan Town, then flows through the main industrial areas, residential areas, and the city center of Liuzhou City. Therefore, the Liujiang River becomes the main waterway of Liuzhou City, in which HMs have been frequently discharged. The upper reaches of the Liujiang River include Duliujiang River, Xunjiang River, Rongjiang River and Longjiang River. Along these rivers, the mining industries are widely distributed, hence, the concentrations of HMs in these rivers are generally high. Luoqingjiang River, the tributary of Liujiang River, originates from Lingui County of Guilin City. Though the watershed of Luoqingjiang River includes mainly the rural areas without significant industrial operation, some industrial parks and part industrial areas in east suburban areas of Liuzhou City also belong to its watershed [6]. The wide distribution of carbonate and karst landforms breed the intact karstic groundwater system in the Liuzhou River Basin, so that the continuous recharge of alkaline groundwater from karstic groundwater system to Liujiang River and Luoqingjiang River comes out, which also keeps the water constantly alkaline in these rivers.

**Figure 1:**
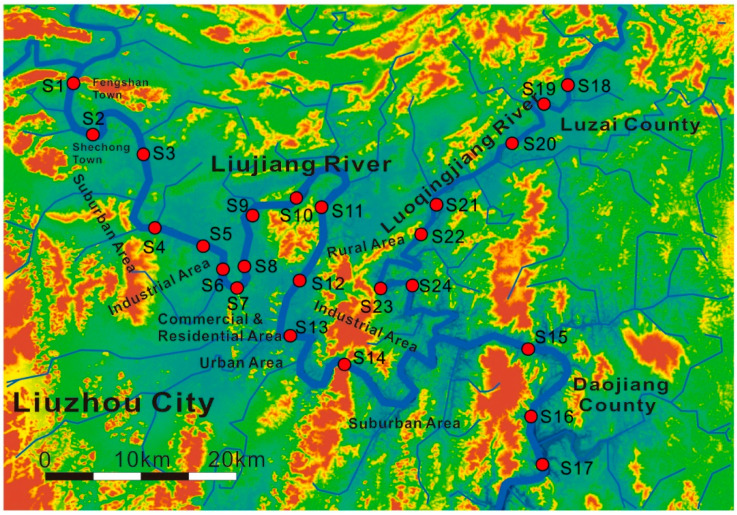
Sampling sites along the Liujiang River.

The sample collection was conducted during May 12th to 18th, 2019, one week after two-week continuous rainfall to avoid the possible weakening of HMs conversion. A total of 24 sampling sites were selected along the Liujiang River and its tributary Luoqingjiang River and a total of 24 surface water and sediment samples were obtained (Figure 1). The sediment samples were collected by using the grab sampler. The overlying water was simultaneously collected to detect the water chemistry (i.e., pH, redox potential (Eh), dissolved oxygen (DO), electrical conductivity (EC), total dissolved solids (TDS), and turbidity) and the content of dissolved HMs. A total of 5 mL nitric acid was added in water samples after being stored in 500 mL polypropylene plastic bottles, while the sediment samples were preserved in polythene self-sealing bags. All of these samples were transported at −20 °C until further processing. 

### 2.2. Sample Preparation and Analysis

The water samples were directly filtered via 0.22 μm syringe-driven filters for further testing.

For sediments, the sediment was freeze-dried to a constant weight at −80 ℃ for 72 h, then sieved through a 0.15 mm nylon mesh. A total of 0.2 g of the sediment samples was digested by a solution of ${\mathrm{HNO}}_{3}+\mathrm{HCl}+\mathrm{HF}$ (5:4:1 *v*/*v*) in a Teflon digestor at 140 ℃ for 6 h. The residue obtained was diluted to make up the volume, then the samples were filtered with 0.22 μm syringe-driven filters for total contents of target HMs testing. 

The distribution of HMs in different chemical forms (exchangeable (Fr1), carbonate-bound (Fr2), reducible (Fr3), oxidizable (Fr4), and residual fractions (Fr5)) were obtained in accordance with the sequential extraction procedure (SEP), which is described in detail in a previous study [33]. To obtain the percent recovery of the HMs using the adopted SEP, the total HMs concentrations were compared with the total concentrations of the HMs in the five SEP-derived fractions. 

Cd, Cr, Cu, Pb, and Zn were analyzed by the inductively coupled plasma mass spectrometry method (ICP-MS, Thermo X series), while Hg and As were measured with the atomic fluorescence spectrometry method (AFS-920). Organic matter was tested by elemental analyzer (Vario EL-III, Elementar, German ), sediment particle size was tested by laser particle size analyzer (Mastersizer 2000, Malvern Instruments, UK).

### 2.3. Quality Assurance and Quality Control

Standard and blank samples were randomly inserted in the test process of each batch of samples. The involved standard substances are: GBW 07401, CRM, GSS-5, SRM2976, which were purchased from the Chinese academy of measurement science. All measurements are minus the average of blank samples as the final sample test values, and a parallel sample was set for each sample, with the average value as the final result. The standard material was used in the sequential extraction process to ensure the accuracy of the extraction results, and the sample recovery ratio was calculated after the test. High sample recovery between 95–105% was reported and used towards QA/QC compliance, and for the detailed analytical limits of detection and quantitation (LOD, LOQ) and the operating parameters of analytical instrumentation refer to [6,34]. For all samples, the total concentrations in the five fractions of HMs were compared with the total HM concentrations, and the average percent recovery was 93.68% ± 5.52%.

### 2.4. Statistical Analysis

The correlations between the data were analyzed by SPSS 20, and *p* < 0.05 indicated significant difference in the data. Figures and tables were finished with Origin Pro 8 (OriginLab, USA) and Coreldraw X4 (Corel, Canada).

### 2.5. Ecological Risk Assessment Method

The potential ecological risk index (RI) is widely used to assess the ecological risks of toxic substances and pollutants to biotas [34,35,36,37]. The calculation formulas of RI are shown below:

(1) $$C_{f}^{i}=\frac{C^{i}}{C_{n}^{i}}$$

(2) $$E_{r}^{i}=T_{r}^{i}\times C_{f}^{i}$$

(3) $$\mathrm{RI}=\overset{n}{\underset{i=1}{\sum }}E_{r}^{i}$$

where: $C^{i}$, $C_{n}^{i}$ and $T_{r}^{i},$ respectively, represent the heavy metal i concentration in sediments (μg/g), the relevant background values of soil in Guangxi Province, and the biological toxic factor of each heavy metal. The soil background values $C_{n}^{i}$ and toxicity factors $T_{r}^{i}$ of HMs can be found in Table S1. The ratio of the measured concentration of the heavy metal in sediment ($C^{i}$) and the corresponding background value ($C_{n}^{i}$) represents the single element contamination factor ($C_{f}^{i}$). $E_{r}^{i}$ is the monomial ecological risk factor of an individual heavy metal, while the sum of $E_{r}^{i}$ is the total ecological risk index (RI). The grade of HM pollution and ecological risk assessment used in this study can be found in Table S2.

## 3. Results and Discussion

### 3.1. The Variations of Water Chemistry in the Surface Water

**Figure 2:**
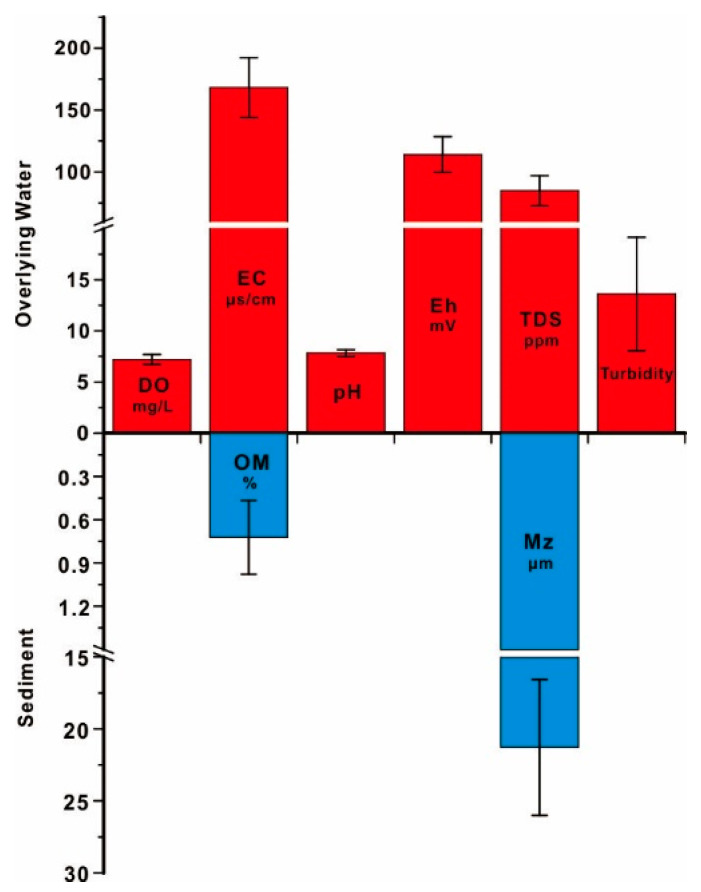
The properties of water and sediment in the Liujiang River Basin.

The variations of water chemistry are given in Figure 2, the relative standard deviations (RSD) of pH and DO are all below 10%, indicating the indistinctive variation of these indexes. The pH generally shows weakly alkaline (with an average of 7.81), which conforms to the characteristics of rivers in karst regions [30]. The faintly acid water was only found in upstream of Liujiang River (pH is 6.9 in S1), which suggested the perturbation of recharge from underground water in karst area. The water is considered to be oxygen-rich in the Liujiang River Basin with the ranges of DO from 6.22 to 8.43 mg/L. The relatively high RSD of Eh, EC, TDS, and turbidity suggest their larger spatial variations. The variation of Eh is 94.89–161.10 mV, while the variation of EC and TDS is 141.5–252.2 and 71.1–127.2, respectively. Turbidity is an index of suspended particulate matter in water, which ranges from 8.13–28.10 NTU, which is higher downstream of the Liujiang River Basin (Refer to Hao, 2021) [32]. 

### 3.2. The Distribution of Particle Size in Surface Sediments

Sediments are mainly clayey silt (Figure S1), among them, silt (4–63 μm) is considered to be the dominant component, the mean proportion of which reach to 74.05%. Clay (<4 μm) is the secondary component of sediments, which holds the ratio of 22.16%. The ratio of sand (63–125 μm) was found to be significantly low in sediments, which is only 3.79%. The grain size of sediments is relatively finer midstream and downstream of Liujiang River (S9 and S10, S13 and S14) and Luoqingjiang River (S23 and S24), which should be blamed on the variation of particulate sources and the sedimentary environment. The average value of OM in surface sediments is 0.72% (Figure 2), which is relatively low, but significant space variation of OM can be found in this study, which is suggested by high RSD of OM (35.42%). 

### 3.3. The Distribution of HMs in Surface Water and Sediments

#### 3.3.1. The Distribution of HMs in Surface Water

**Figure 3:**
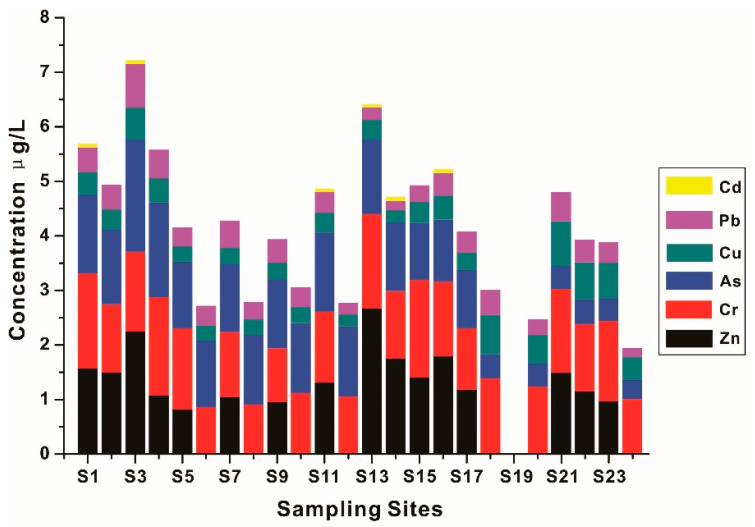
HMs concentrations in the surface water.

The concentrations of HMs in surface water are given in Figure 3 and Table S3. The contents of Hg in water samples are all below the detection limit, while the contents of other HMs decrease with the order: Zn > Cr > As > Cu > Pb > Cd > Hg. HMs are all within the primary standard of national surface water quality released by State Environmental Protection Administration of China (GB 3838–2002) [38], suggesting that the concentrations of HMs in surface water are generally not high and the strictly controlled sewage into the river in recent years has already exerted a positive effect in watershed of the Liujiang River. For Liujiang River, the contents of HMs in water decrease from upstream to downstream, the higher HMs concentrations mainly centralize in upstream (S1–S4), which should be attributed to the ongoing operation of prosperous mining in Longjiang River and Duliujiang River in the upper reach of Liujiang River [39,40,41]. The concentrations of HMs gradually decrease with the river flowing downstream, but the lowest contents of HMs are observed only in midstream (S5–S12), where the main urban areas are located in. The HM concentrations slightly increase downstream of the Liujiang River (S13–S17), which should be blamed on some pollution industries moving to suburban areas. In addition, the HM concentrations in Luoqingjiang River are overall lower than that in the Liujiang River, which approach those in the midstream of the Liujiang River and are also related to the lower distribution of pollution industries.

#### 3.3.2. The Distribution of HMs in Sediments

**Figure 4:**
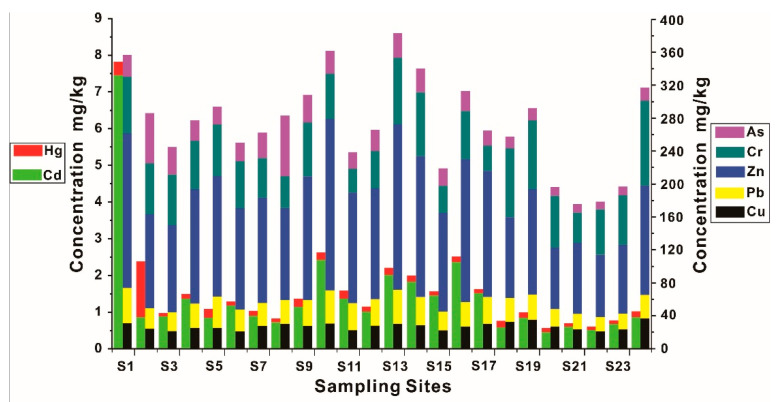
The distribution of HMs in surface sediments.

The distribution of HM contents in sediments is shown in Figure 4. The concentrations of HMs are in the decreasing order as follows: Zn > Cr > Pb > Cu > As > Cd > Hg. Among them, most HMs were between TEL (Threshold Effect Level) and PEL (Probable Effect Level) (Table S4), suggesting a low risk exerting negative effects on the benthos, while Cu and Pb are usually lower than TEL. For the soil background of Guangxi (Table S4), most HMs were close to or lower than their corresponding soil baseline, but Zn and Cd were significantly higher, which suggested a potential interference from human activities. The distribution of HMs in sediments is found to be similar with that in water, most HMs are higher in upstream of Liujiang River (S1 and S2). In particular, the concentration of Cd at S1 is 5.3 times higher than the average value. This further indicates that there is still a large amount of Cd remaining upstream of the Liujiang River in spite of the notorious Cd pollution event passing 8 years [40]. The concentrations of HMs are relatively low in midstream of the Liujiang river (S3~S9), while the concentrations rebound in the downstream (S10~S17). The contents of HMs are overall lower in Luoqingjiang River (S18~S24) than those in Liujiang River.

#### 3.3.3. The Speciation of HMs in Sediments

**Figure 5:**
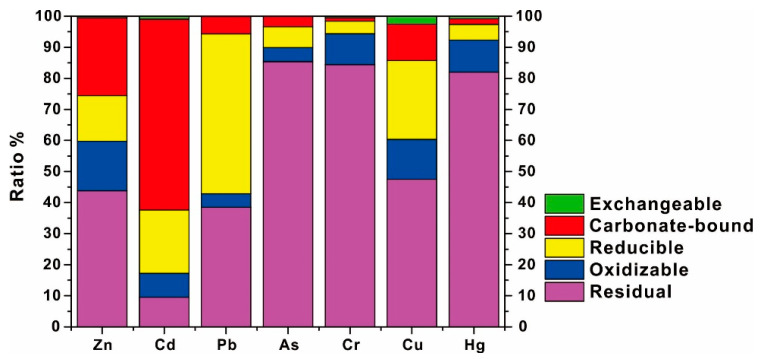
The chemical forms of HMs in surface sediments.

The speciation of HMs in sediments is shown in Figure 5. HMs are mostly in the residual fraction, indicating that they are derived from crustal materials and less likely involve the biogeochemical circle of HMs due to the inertia of the minerals containing HMs [20]. The ratio of As, Cr, and Hg in the residual fraction is extremely high (over 80%), which strongly indicates a natural source, while the proportions of Cu and Zn in the residual fraction are less than 50%, which suggests a certain amount of anthropogenic input. For Pb and Cd, their residual fractions are not the dominant fraction, and their dominant fractions are, respectively, reducible and carbonate-bound fractions, which expresses an intense exogenous input from human activities.

For non-residual fractions, the proportions of exchangeable HMs are extremely low, indicating that the exchangeable fraction is not the main speciation of HMs and also should be attributed to the low contents of HMs in water. Zn and Cd are primarily in the carbonate-bound fraction, which suggests they are primarily weakly reserved in sediments. Pb, Cu, and Cd are relatively high in reducible fractions, implying Fe/Mn oxy-hydroxides play a vital role in stabilizing Pb, Cu and Cd. Due to the shortage of organic matter in sediments, organic matter fails to effectively compete with Fe/Mn oxy-hydroxides, and then directly lowers their combination with HMs, so that the proportions of HMs in the oxidizable fraction were commonly lower than 15%. 

The significant correlations of HMs between the total contents and various forms are found in this study (Table S5), indicating that the elevation of HM concentration would have a vital impact on the speciation of HMs. For Cr, Cu, As and Hg, the correlations between total contents and residual fraction are found to be more significant, suggesting that the elevation of their residual fraction would be a priority with their total contents increasing, so that their increasing in total contents would be hard to affect their stability and bioavailability in sediments. For Zn, Pb, and Cd, the correlations were found to be more significant with the non-residual fraction (respectively, carbonate-bound, reducible and oxidizable fraction), instead of the residual fraction, suggesting that their non-residual fraction will be increasing in priority with their total content elevation, in other words, the elevations of these HMs are dominated by exogenous input. Given the high mobility and toxicity of HMs in the non-residual fraction [20,21], it is, thus, essential to be vigilant on the excessive input of exogenous Zn, Pb, and Cd in the Liujiang River Basin.

### 3.4. Accumulation Mechanism of HMs in Surface Sediments of Liujiang River Basin

#### 3.4.1. The Existing Forms of HMs in Surface Water

The results of correlation analysis between hydrochemistry and HMs in water were shown in Table 1. The correlations of HMs are all found to be non-significant with pH, Eh, indicating that pH and Eh are trivial in elevating the solubility of HMs in river. On the contrary, the correlations between HMs and other hydorchemical factors, i.e., EC, TDS, turbidity and DO, are found to be significant, which expresses the existing forms of HMs in water. Zn and Cr are significantly correlated with EC and TDS, indicating that Zn and Cr exist as dominant ions in water, which is obviously not consistent with the fact that HMs are just trace ions in water, and the excessive existence of Zn and Cr in water, thus, should be considered to be the result of anthropogenic emissions. Due to the higher bioavailability of dissolved HMs, the excessive existence of exogenous Zn and Cr in water must elevate their bio-enrichment among aquatic biotas, which was well expressed in our previous studies that Zn and Cr in wild fish are mostly beyond maximal residual limited [6]. The significant correlation of Cu is found to be positive with turbidity, indicating that Cu is mainly in the form of particles, so that exogenous Cu may be discharged mainly in granular form. There is a significant positive correlation between Cd and DO, which indicates the dissolubility of Cd can be elevated with DO increasing. Despite the fact that exogenous emission of Pb is confirmed in this study, the correlations between Pb and hydrochemical parameters are not found, which maybe suggest the limited emission of Pb. Arsenic is determined mainly from natural sources in this study, so that As in water may be close to the background value. The significant correlation of As was only found to be negative with turbidity, indicating the suspended particles have a dilution effect on As in water.

#### 3.4.2. Impact Factors of the Accumulation of HMs in Surface Sediments

The composition of particles size and organic matter have a vital impact on the accumulation of HMs in sediments [41,42]. As shown in Table 2, the significant correlations between HMs and particle size are found to be negative. Fine particles have a strong binding capacity to HMs, which is not only related to their higher specific surface area, but also higher concentration of active components in fine particles, i.e., organic matter, carbonate, Fe/Mn oxides, etc., all of which can further facilitate the absorption of HMs in sediments [34]. The significant positive correlations between organic matter and HMs demonstrate that the increase of organic matter can significantly increase the concentrations of HMs in sediments, which is related to the combination of exogenous HMs and organic matter [20]. However, the significant correlations of Cd, As, and Hg cannot be found with grain size and organic matter. For Cd, the effects of grain size and organic matter may be relieved by the large input of exogenous Cd. The effects of grain size and organic matter is non-significant on As and Hg, mainly due to their less exogenous input and relatively uniform distribution in sediments.

The more significant correlations of HMs can be only found with Eh and turbidity rather than other hydrochemical factors, suggesting that Eh and suspended particles should be treated as the manipulators of HMs accumulation in rivers. It is well-known that higher Eh promotes the formation of Fe/Mn oxides in sediments, which will aggravate the aggregation of exogenous HMs [32]. That explained the positive significant correlation between Zn, Pb, Cd and Eh. For turbidity, the significant correlation is found to be negative with Zn, Pb and Cu, indicating that these HMs eventually enter the sediments under the mediation of suspended particulate matter, regardless of the dissolved or particulate form they discharged. Given that Zn, Pb, Cd and Cu are all higher in reducible phase, we, thus, have a reason to believe that the regulation of hydrochemistry may be greater on HMs with higher content in the reducible phase. Therefore, the ecological threat resulting from higher concentration of HMs in the reducible phase should not be ignored. However, for Cr, there are no significant correlations with turbidity and Eh, indicating that exogenous Cr is not easy to be absorbed by suspended particles, which results in the limited input of exogenous Cr in sediments and the high proportion of Cr in the residual phase. Therefore, exogenous Cr may exist mainly in the water body and less in the sediments.

pH fluctuation is an important factor that activates the HMs in sediments [42,43]. However, except for Cd, the significant correlations of pH cannot be found with HMs in this study, indicating that pH maybe a trivial factor in HM accumulation in river, while the significant correlation of Cd is negative with pH, which is obviously inconsistent with the traditional understanding that alkaline condition promotes the aggregation of HMs, but resulting from sewage discharging. In fact, the industries in this study area are dominated by electroplating, steel production and machinery manufacturing [6], the sewage from these industries is mainly acidic and the concentration of Cd in sewage could be superior. Therefore, the large emission of the sewage will elevate the input of exogenous HMs in sediment, while the large emission of the sewage will reduce the pH of water at the same time. The positive significant correlation between EC and Cd also confirms the input of exogenous Cd in sediments, the aggregation of Cd in sediment is found to be elevated with ions increasing, instead of diluting. This explains that why the decrease of pH does not destabilize the aggregation of Cd in sediments, but significantly increases the concentration of Cd in sediments. Therefore, it is reasonable to believe that Cd in sediments is dominated by exogenous input, so that the influence of pH fluctuation on the accumulation of Cd in sediments was reversed by sewage discharging. As and Hg are mainly from natural sources, with a high proportion of residual fraction, so that they are insensitive to changes in water conditions.

#### 3.4.3. Controlling Factors on HM Speciation in Surface Sediments

In order to further understand the specific mechanism of HM accumulation in sediments, the correlations analysis between different forms of HMs and various factors are shown in Table S6. In general, a large input of exogenous HMs can not only change the influences of hydrochemical fluctuations on the speciation of HMs in sediments, but also can reverse the influences of hydrochemical fluctuations on the speciation distribution of HMs in sediments [32], which is well-reflected in Cd. The increase of EC did not aggravate the competition of ions to reduce the content of Cd in the non-residual phase [22], but intensified the accumulation of Cd in all non-residual phases, which directly elevates the mobility and bioavailability of Cd. The significant degree of elevation disclosed by the correlation coefficients between EC and Cd in different forms, which decreased in the order: carbonate-bound fraction, reducible fraction, oxidizable fraction and exchangeable fraction, and this sequence is exactly consistent to their respective contents in different species of HMs (Table S7), also suggesting the order of exogenous Cd that combined with active substances in sediments. Besides Cd, the negative correlations of pH were also found to be significant with Cu and Hg in the exchangeable phase and oxidizable phase, indicating the discharge of acidic wastewater can also increase the contents of these HMs in these forms, therefore, more strict controlling on wastewater discharging in Liujiang River Basin should be considered to be a critical approach that lowers the aggregation of Cd, Cu and Hg in non-residual forms.

According to the previous analysis in this study, the accumulation of Fe/Mn oxides is conducive to the aggregation of exogenous HMs in sediments [21], which is also well-reflected in the speciation of HMs. The significant correlations between Eh and the speciation of most HMs indicate that the elevation of Eh can promote the accumulation of exogenous HMs in other non-residual fractions, but the higher significance indicates that the increase of Eh has a more important influence on the reducible phase and carbonate-bound phase, which highlights the crucial role of Eh on the stability of HMs in sediments. There are significant positive correlations between the DO and Cr in carbonate-bound phase, reducible phase and Zn in oxidizable phase, indicating that the high DO content is beneficial to the accumulation of these forms. In fact, DO is the important index of photosynthesis [44], the stronger photosynthesis will elevate the content of the dissolved oxygen and consume more carbon dioxide, eventually decreasing the acidity in river, which is conducive to the stabilization of HMs in the carbonate-bound phase. In addition, the high dissolved oxygen content is conducive to the accumulation of Fe/Mn oxides and promotes the aggregation of HMs in the reducible phase, while organic compounds that are synthesized by photosynthesis can contribute to the accumulation of HMs in the oxidizable phase [30]. Therefore, although the correlation of dissolved oxygen is found to be non-significant with total concentrations of HMs, its impact on the accumulation of HMs in some forms exists. The correlations of turbidity are found to be negative with all forms of HMs, including residual fraction, indicating that the input of HMs mediated by suspended particles is not limited to the exogenous, but also the endogenous. For the physicochemical properties of sediments, coarse particles can significantly reduce the contents of various forms of HMs, while the impacts of organic matter are totally opposite. Regardless of HM forms, they are all preferred to aggregate in clay, instead of sand and silt.

#### 3.4.4. The Conversion and Accumulation of HMs between Sediments and Water

The correlations of HMs between surface water and surface sediments are shown in Table 3. Most HMs are high in the residual fraction, therefore, the non-significant conversion of HMs between water and sediments should be blamed on the relatively limited exogenous input of most HMs, which is just confirmed by the fact that the significant correlation of most HMs in water is not found with those in sediments. However, the significant correlations of most HMs in some chemical forms are found to be correlated with HMs in water, suggesting some chemical forms play a vital role in regulating the conversion of HMs. The significant correlations between water and sediments are found to be Cd, As, Cu, Zn and Pb, but only the Cd is found to have the significant correlation in all non-residual fractions. The significant correlations of Cd between all non-residual fractions in sediment and surface water are found to be positive, which indicates that the large emission of wastewater elevated all non-residual fractions of Cd in sediments, however, the correlation coefficients of each non-residual fraction are relatively even, which suggested that the emission of Cd from wastewater was high enough to mitigate the preference of Cd combination with Fe/Mn oxide, organic matter and carbonate. Therefore, it is urgently needed to relieve the risk of Cd pollution in the Liujiang River Basin by reducing the emission of wastewater. 

Similar to Cd, the significant correlation of Zn between water and sediments is found to be positive with the reducible phase, indicating that the ions of Zn that discharged from wastewater will mainly aggravate the aggregation of Zn in the reducible phase with the impacts of the strong binding force between Fe/Mn oxides and Zn [21]. However, the correlations of Cu and Pb, which are different to Zn, are found to be negative in the oxidizable phase and in residual phase, respectively. For Cu, the significant correlation suggested that the oxidizable fraction in sediments should be treated as the main destination of exogenous Cu, therefore, the binding of exogenous Cu with organic matter and sulfide should be considered to be the main way to purify water. For Pb, the significant correlation expresses that the natural deposition of endogenous Pb has a significant dilution effect on the accumulation of exogenous Pb.

For Cr, the correlation of Cr in water was found to be non-significant with that in any forms and total content in sediment, which is obviously inconsistent with the existence of the exogenous Cr in water. Actually, the water is commonly found to be alkaline in this study, while the exogenous Cr was confirmed to be more likely to remain in water under alkaline conditions [45], that is the reason why the ratio of Cr in non-residual fraction (16%) fails to rise with the existence of the exogenous Cr in water. Despite the fact that the concentrations of Hg in water are all below detection limit, the extremely high ratio of Hg in residual phase similar to Cr also suggests that the accumulation of Hg is dominantly from natural deposition, while the aggregation of exogenous Hg in sediments from water may not be significant. 

Although HMs in the non-residual fraction play a critical role in regulating the accumulation of exogenous HMs in the aquatic environment, HMs in the residual fraction can also affect the conversion of HMs between sediment and water, especially when microorganisms are involved. As was found to be the best instance in this study, which is dominant in the residual fraction, the correlation of As between water and sediments should have been nonsignificant, however, a significant correlation is observed. The significantly positive correlations of As in water with that in carbonate-bound and reducible phases suggest that carbonate and Fe/Mn oxides have an important role in the fixation of exogenous As. However, the significantly positive correlation of As in the residual phase indicates that the inert endogenous As can also impact water quality. In fact, the reductive dissolution of Fe/Mn oxide or minerals manipulated by microbes can even release the As contained in minerals [46,47], which indicates that As has a vital influence on water quality, regardless of that in non-residual or residual fractions.

### 3.5. Potential Ecological Risk Assessment of HMs

**Figure 6:**
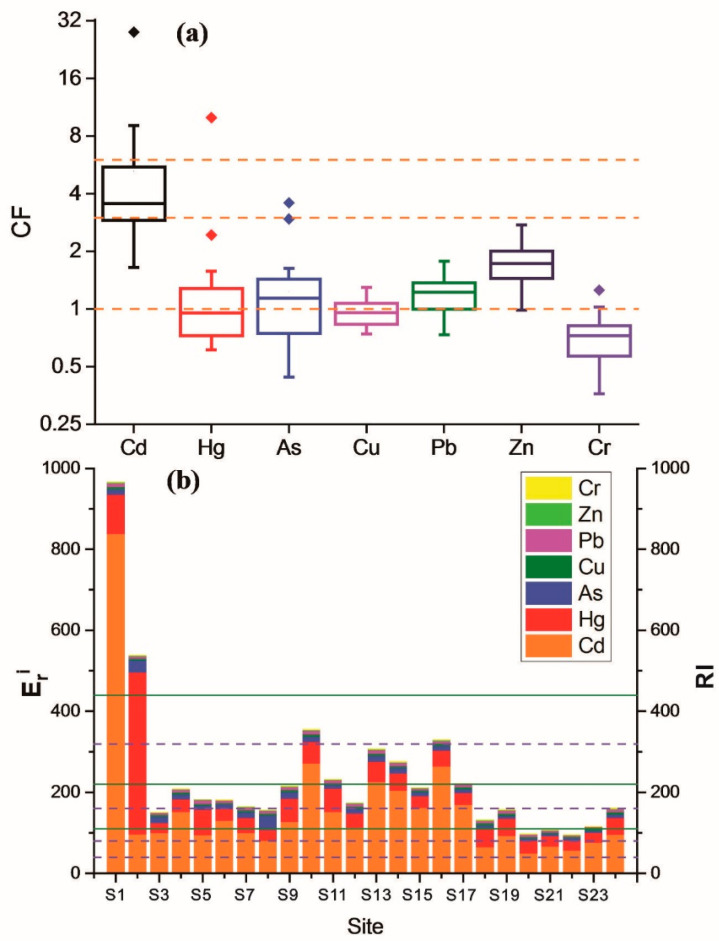
Risk assessments for the HMs in surface sediments: (**a**) Box plots of contamination factors of the HMs; (**b**) $E_{r}^{i}$ and RI of the HMs in different sampling positions. The dotted line and the solid line represent the grade of $E_{r}^{i}$ And RI, respectively.

The assessments of HM contamination in sediments are shown in Figure 6. Except Cd, the CF values of HMs are almost all below 3, suggesting low-to-moderate pollution level. The mean CF of Hg, Cu and Cr are all below 1, expressing low pollution level, while the mean CF of Zn, Pb and As is within 1 to 3, indicating moderate pollution level. The CF of Cd is significantly higher and commonly beyond 3, but below 6, suggesting a considerable pollution level. The maximum $E_{r}^{i}$ of Cd appears in the upper reach of Liujiang River (S1), reaching a very high risk, while S10 and S13~S17 in the lower reach of Liujiang River present a high risk, and Luoqingjiang River shows a moderate risk overall. For Hg, the average of $E_{r}^{i}$ is 56, presenting a moderate risk overall. The maximum value appears at S2 in the upper reach of Liujiang River, which reaches very high risk, while the rest shows low or moderate risk.

The average of RI reached 239, suggesting that the study area is generally with moderate risk. The RI values of S1 and S2 were high, which obviously exceed the limits of very high risk and considerable risk, respectively. The ecological risks of other sampling sites are close to or lower than the moderate risk. The RI of Luoqingjiang River is obviously lower, presenting low risk. In general, the potential ecological risk of HMs of surface sediments in the Liujiang River is higher than that in the Luoqingjiang River. The HM risk of the Liujiang River decreases gradually from upstream to downstream, but slightly rebounds downstream. Cd has a higher risk than other HMs. Therefore, Cd pollution of sediments in the Liujiang River Basin should be paid more attention.

## 4. Conclusions

The obtained results suggested the concentrations of HMs in water are far below the primary standard of water quality, but in sediments, the contents of Cd and Zn are significantly higher than their corresponding baseline of soil. Cd and Pb are mainly in the carbonate-bound fraction and reducible fraction, respectively, while other HMs are mainly in residual fraction. The non-significant correlations suggested pH and Eh may be hard to influence HMs in water, while the significant correlations highlighted the regulations of Eh, organic matter and mean grain size on the accumulation of metals in sediments. The opposite correlations between EC, TDS, pH and Cd confirmed the emission of acid wastewater contributed to the accumulation of Cd in sediment. The conversion of metals between water and sediments was found to be significant only in the specific chemical form of Cd, As, Cu, Zn and Pb, suggesting the conversion of HMs in sediments should be largely regulated by their specific chemical forms, so that cutting down the accumulation of HMs in specific forms should be considered as an effective approach to degrade their conversion. The very high risk disclosed by the higher values of $E_{r}^{i}$ and RI are only found upstream, while the higher risk of Cd should be treated as a critical environmental threat. 

## Figures and Tables

**Table 1:** The correlations of HMs in surface water and hydrochemical characteristics.

	DO	EC	pH	Eh	TDS	Turbidity
**Cu**	−0.096	−0.148	−0.358	−0.280	−0.148	0.648 **
**Pb**	−0.181	−0.053	−0.168	−0.089	−0.054	0.181
**Zn**	0.400	0.408 *	0.177	−0.130	0.407 *	−0.136
**Cr**	0.254	0.429 *	−0.114	0.041	0.428 *	0.134
**Cd**	0.480 *	0.232	0.076	0.224	0.232	−0.272
**As**	0.307	0.128	0.290	0.271	0.127	−0.486 *

* Correlation is significant at *p* < 0.05; ** Correlation is significant at *p* < 0.01. Correlation is significant in bold. The concentrations of Hg are all below the detection limit.

**Table 2:** The correlations between HMs in sediments and environmental factors.

	DO	EC	pH	Eh	TDS	Turbidity	MZ	OM
**Cu**	0.163	0.189	−0.084	0.093	0.192	−0.415 *	−0.666 **	**0** .889 **
**Pb**	0.381	0.286	0.032	0.500 *	0.286	−0.659 **	−0.404	0.454 *
**Zn**	0.347	0.295	0.083	0.459 *	0.295	−0.612 **	−0.424 *	0.331
**Cr**	0.242	0.272	−0.103	−0.214	0.272	−0.167	−0.579 **	0.486 *
**Cd**	0.109	0.732 **	−0.501 *	0.739 **	0.729 **	−0.321	−0.045	0.025
**As**	0.078	−0.200	0.169	0.236	−0.200	−0.379	0.156	−0.225
**Hg**	0.075	−0.061	−0.031	−0.134	−0.060	−0.226	0.183	−0.163

* Correlation is significant at *p* < 0.05; ** Correlation is significant at *p* < 0.01. Correlation is significant in bold.

**Table 3:** The correlations of HMs between surface water and sediments.

	WCr	WCu	WZn	WCd	WPb	WAs	WHg
**Fr1**	0.096	0.275	−0.012	0.620 **	−0.280	0.232	-
**Fr2**	0.026	−0.330	0.312	0.513 *	−0.101	0.491 *	-
**Fr3**	0.163	0.068	0.448 *	0.526 **	−0.312	0.631 **	-
**Fr4**	−0.049	−0.427 *	0.400	0.577 **	−0.400	0.397	-
**Fr5**	0.007	−0.330	−0.004	0.394	−0.477 *	0.515 *	-
**Total**	0.010	−0.274	0.286	0.524 *	−0.356	0.522 **	-

* Correlation is significant at *p* < 0.05; ** Correlation is significant at *p* < 0.01. Correlation is significant in bold

## Data Availability

The original contributions presented in the study are included in the article, further inquiries can be directed to the corresponding author.

## Supplementary Materials

The following supporting information can be downloaded at: https://www.mdpi.com/article/10.3390/ijerph19031619/s1, Table S1: Soil background values and toxicity factors of HMs; Table S2: Ecological risk assessment rating; Figure S1: The composition of grain size and mean particle size in surface sediments; Table S3: The concentrations of HMs (μg/L) in surface water and their respective thresholds in primary standard of environmental quality standards for surface water in China; Table S4: Sediment Quality Guidelines and baseline of HMs (mg/kg); Table S5:　The concentrations of HMs (μg/L) in surface water and their respective thresholds in primary standard of environmental quality standards for surface water in China (SEPA, 2002); Table S6: The correlations between the total concentrations of HMs and their forms; Table S7: The speciations of heavy metal in surface sediments (%); Table S8: The correlations between chemical forms of HMs and environmental factors.
