# Supplementary material for: The Accumulation and Transformation of Heavy Metals in Sediments of Liujiang River Basin in Southern China and Their Threatening on Water Security
Source: Int J Environ Res Public Health. 2022 Jan 31;19(3):1619. doi: 10.3390/ijerph19031619 (PMC8834996; doi:10.3390/ijerph19031619)
Supplement: Supplementary file 1 [file ijerph-19-01619-s001.zip › ijerph-1496629-supplementary.pdf]

The Accumulation and Transformation of Heavy Metals in Sediments of Liujiang River in Southern China and Their Threatening on Water Security

Xiongyi Miao [1,2,3], Mian Song [5], Gaohai Xu [6], Yupei Hao [1,4,*] and Hucai Zhang [4,*]

[1] Key Laboratory of Karst Dynamics, MNR&GZAR, Institute of Krast Geology, Chinese Academy of Geological Sciences, Guilin 541004, China

[2] Department of Health Management, Guiyang Healthcare Vocational University, Guiyang 550001, China

[3] Henan Xinweijie Technology Co., Ltd., Luoyang 471000, China

[4] Institute for Ecological Research and Pollution Control of Plateau Lakes, School of Ecology and Environmental Science, Yunnan University, Kunming 650500, China

[5] Center for Hydrogeology and Environmental Geology, CGS, Baoding 071051, China

[6] Nanjiang Hydrogeological & Engineering Geology Brigade, Chongqing Bureau of Geology and Minerals Exploration, Chongqing 401121, China

Supplementary Materials

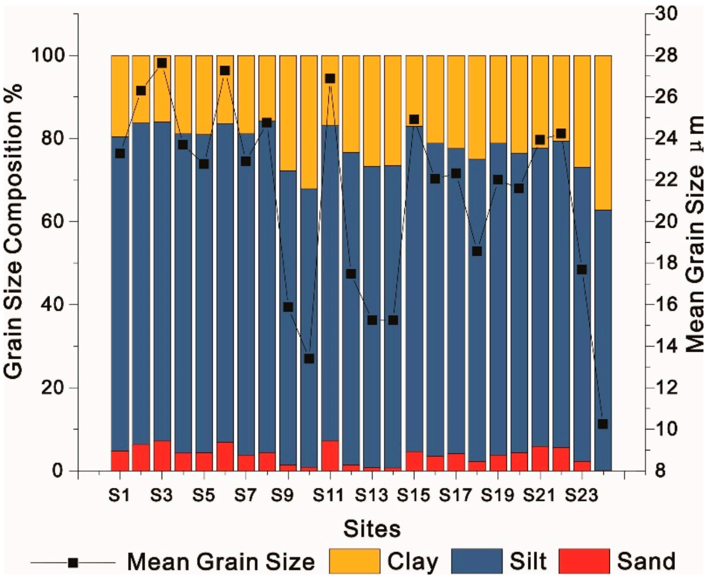

Figure S1. The composition of grain size and mean particle size in surface sediments.

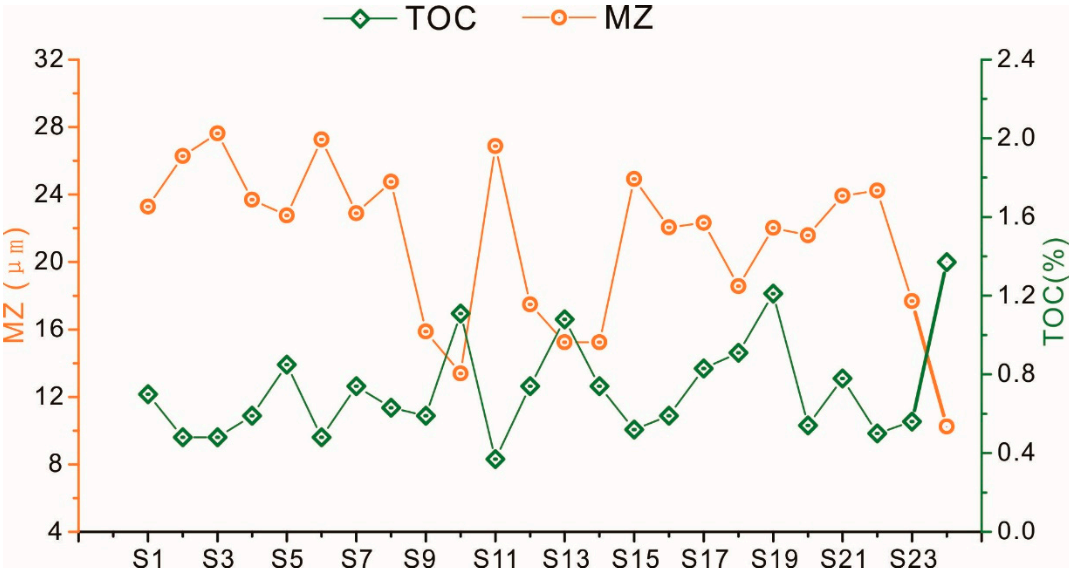

Figure S2. The distribution of TOC and mean particle size in surface sediments.

Table S1

Soil background values and toxicity factors of HMs (CNEMC, 1990; Hakanson, 1980; Liu et al., 2017).

Item	Cr	Cu	Zn	Pb	Cd	As	Hg
C_n^i(mg/kg)	82.1	27.8	75.6	24	0.267	20.5	0.152
T_r^i	2	5	1	5	30	10	40

Table S2

Ecological risk assessment rating (Hakanson, 1980; Liu et al., 2017; Miao et al., 2019c).

Grade	C_f^i(CF)	Pollution level
1	< 1	Low
2	1-3	Moderate
3	3-6	Considerable
4	>6	Very high
**Grade**	E_r^i	**Ecological risk level**
1	< 40	Low
2	40-80	Moderate
3	80-160	Considerable
4	160-320	High
5	> 320	Very high
**Grade**	**RI**	**Ecological risk level**
1	< 110	Low
2	110-220	Moderate
3	220-440	Considerable
4	> 440	Very high

Table S3

The water chemistry in surface water.

	DO(mg/L)	EC(μs/cm)	pH	Eh(mV)	TDS(ppm)	Turbidity
**min**	6.22	141.5	6.79	94.89	71.1	8.13
**max**	8.43	252.2	8.48	161.10	127.2	28.10
**mean**	7.20	168.1	7.81	114.03	84.9	13.61
**RSD**	6.77%	14.32%	4.57%	12.77%	14.33%	40.75%

Table S4

Sediment Quality Guidelines of HMs (mg/kg) (Chapman et al., 1999; Smith et al., 1996).

	Cd	Pb	Cr	Cu	Zn	As	Hg
**TEL**	0.6[a]	35[a]	37[a]	36[a]	123[a]	8.2[b]	0.17[a]
**PEL**	3.5[a]	91[a]	90[a]	197[a]	315[a]	70[b]	0.49[a]

Note: a. The freshwater sediment quality of Canada; b. The sediment quality criteria of Hong Kong.

Table S5

The concentrations of HMs (μg/L) in surface water and their respective thresholds in primary standard of environmental quality standards for surface water in China (SEPA, 2002).

	Cu	Pb	Zn	Cr	Cd	As	Hg
**Range**	0.19-0.82	0.17-0.80	BDL-2.67	0.86-1.81	BLD-0.08	0.35-2.05	BDL
**Mean**	0.41	0.39	1.45	1.33	0.07	1.11	BDL
**Primary Standard**	10	10	50	10	1	50	0. 05

Note: BDL means below the detection limit.

Table S6

The correlations between the total concentrations of HMs and their forms.

	Cr	Cu	Zn	Cd	Pb	As	Hg
**Fr1**	**0.699****	**0.669****	**0.529****	**0.701****	0.310	0.168	**0.862****
**Fr2**	0.406	0.411	**0.951****	**0.998****	**0.807****	**0.644****	0.192
**Fr3**	**0.437***	**0.844****	**0.922****	**0.986****	**0.987****	**0.901****	0.144
**Fr4**	**0.681****	**0.542****	**0.863****	**0.877****	**0.931****	**0.919****	0.150
**Fr5**	**0.996****	**0.913****	**0.828****	0.372	**0.591****	**0.998****	**0.983****

[*] Correlation is significant at $p < 0.05$; [**] Correlation is significant at $p < 0.01$.

Table S7

The speciations of heavy metal in surface sediments (%).

	Exchangeable		Carbonate-bound		Reducible		Oxidizable		Residual	
	**Range**	**Average**	**Range**	**Average**	**Range**	**Average**	**Range**	**Average**	**Range**	**Average**
**Zn**	0.26-0.84	0.47	17.04-33.07	25.08	7.79-19.59	14.68	12.56-20.62	15.93	33.72-58.31	43.83
**Cd**	0.44-1.80	0.97	46.71-77.30	61.43	15.28-24.93	20.38	2.19-13.01	7.69	1.66-18.22	9.54
**Pb**	0.01-0.46	0.15	1.87-8.06	5.53	40.99-63.52	51.47	3.75-4.97	4.34	26.65-52.17	38.52
**As**	0.02-0.15	0.08	1.06-9.50	3.28	3.67-9.30	6.74	1.67-12.44	4.52	76.75-90.67	85.38
**Cr**	0.31-0.90	0.53	0.56-1.66	1.04	1.83-6.42	4.05	5.98-17.13	9.95	74.36-90.57	84.43
**Cu**	0.98-4.83	2.54	5.96-17.94	11.7	18.67-33.75	25.41	9.92-17.67	12.83	41.41-54.00	47.51
**Hg**	0.23-1.28	0.82	0.18-2.52	1.76	0.52-8.67	5.09	1.05-64.83	10.34	30.16-98.02	81.98

Table S8

The correlations between chemical forms of HMs and environmental factors.

		MZ	Sand	Silt	Clay	TOC	DO	EC	pH	Eh	TDS	Turbidity
Fr1	Cr	**-0.604****	**-0.513***	**-0.664****	**0.666****	**0.707****	0.095	0.138	-0.089	-0.143	0.138	-0.067
	Cu	-0.372	-0.266	**-0.564****	**0.498***	**0.592****	-0.223	0.176	**-0.418***	-0.159	0.178	-0.014
	Zn	**-0.495***	**-0.508***	**-0.412***	**0.490***	0.349	0.026	0.007	-0.062	-0.009	0.008	-0.074
	Cd	-0.059	-0.141	0.09	-0.006	0.057	0.054	**0.433***	-0.268	**0.494***	**0.432***	-0.327
	Pb	**-0.528****	**-0.519****	**-0.477***	**0.539****	0.33	-0.019	-0.092	-0.032	-0.152	-0.091	0.087
	As	-0.09	-0.153	0.072	0.012	0.174	-0.246	-0.161	-0.026	0.366	-0.161	-0.344
	Hg	0.178	0.212	0.15	-0.189	-0.083	0.238	0.286	-0.201	0.099	0.285	-0.309
Fr2	Cr	**-0.582****	**-0.661****	-0.209	**0.410***	0.377	**0.501***	0.135	**0.435***	0.047	0.135	-0.314
	Cu	-0.031	-0.181	0.248	-0.1	0.16	-0.055	-0.316	0.342	0.099	-0.315	-0.073
	Zn	-0.31	-0.374	-0.064	0.195	0.302	0.195	0.259	0.067	**0.509***	0.258	**-0.501***
	Cd	-0.018	-0.035	0.054	-0.023	0.033	0.106	**0.740****	**-0.509***	**0.746****	**0.737****	-0.306
	Pb	0.049	-0.046	0.331	-0.211	-0.017	0.273	0.15	0.112	**0.636****	0.148	**-0.586****
	As	0.04	-0.079	0.34	-0.204	0.007	-0.067	-0.294	0.234	0.338	-0.294	-0.382
	Hg	-0.054	-0.079	0.091	-0.031	0.165	0.253	**0.596****	-0.337	**0.525***	**0.596****	**-0.420***
Fr3	Cr	**-0.625****	**-0.613****	**-0.442***	**0.552****	**0.505***	**0.442***	**0.460***	0.172	0.105	**0.460***	-0.358
	Cu	**-0.602****	**-0.595****	**-0.517****	**0.597****	**0.870****	0.067	0.319	-0.318	0.052	0.321	-0.209
	Zn	-0.308	-0.335	-0.136	0.228	0.152	0.316	0.375	-0.018	**0.420***	0.373	**-0.482***
	Cd	-0.121	-0.129	-0.054	0.089	0.116	0.09	**0.692****	**-0.492***	**0.704****	**0.690****	-0.332
	Pb	-0.335	-0.371	-0.083	0.206	0.401	0.333	0.295	-0.026	**0.526****	0.295	**-0.620****

	**As**	0.132	0.01	**0.448***	-0.315	-0.113	0.111	-0.075	0.124	**0.511***	-0.076	-0.41
	**Hg**	0.13	0.174	0.039	-0.097	0.011	-0.099	0.193	-0.366	0.326	0.193	0.04
	**Cr**	**-0.747****	**-0.704****	**-0.670****	**0.747****	**0.845****	0.11	0.355	-0.163	-0.008	0.357	-0.151
	**Cu**	-0.197	-0.302	0.154	0.014	0.348	0.344	0.35	0.065	0.321	0.351	**-0.491***
	**Zn**	-0.292	-0.33	-0.053	0.169	0.238	**0.499***	0.254	0.228	0.241	0.255	**-0.650****
**Fr4**	**Cd**	-0.148	-0.159	-0.025	0.081	0.069	0.22	**0.606****	-0.28	**0.589****	**0.604****	**-0.460***
	**Pb**	**-0.476***	**-0.499***	-0.204	0.341	**0.483***	0.392	0.183	0.1	0.355	0.184	**-0.737****
	**As**	0.394	0.334	**0.505***	**-0.484***	-0.314	-0.027	-0.284	0.117	0.027	-0.284	-0.212
	**Hg**	0.071	0.085	0.06	-0.075	0.008	0.009	**0.778****	**-0.640****	**0.666****	**0.776****	-0.16
	**Cr**	**-0.526****	**-0.466***	**-0.482***	**0.522****	**0.508***	0.228	0.242	-0.108	-0.234	0.242	-0.149
	**Cu**	**-0.718****	**-0.666****	**-0.619****	**0.696****	**0.815****	0.196	0.11	-0.03	0.016	0.114	**-0.440***
	**Zn**	**-0.580****	**-0.597****	-0.299	**0.446***	**0.610****	**0.429***	0.213	0.11	0.365	0.214	**-0.679****
**Fr5**	**Cd**	-0.37	-0.315	-0.264	0.309	0.382	0.327	0.256	0.062	0.288	0.256	**-0.517***
	**Pb**	**-0.691****	**-0.706****	**-0.438***	**0.586****	**0.682****	**0.443***	0.201	0.204	0.091	0.202	**-0.505***
	**As**	0.118	0.016	0.396	-0.281	-0.136	0.096	-0.183	0.171	0.236	-0.184	-0.389
	**Hg**	0.172	0.203	0.149	-0.185	-0.162	0.074	-0.204	0.086	-0.258	-0.203	-0.199
